# Numbers needed to treat calculated from responder rates give a better indication of efficacy in osteoarthritis trials than mean pain scores

**DOI:** 10.1186/ar2394

**Published:** 2008-04-02

**Authors:** R Andrew Moore, Owen A Moore, Sheena Derry, Henry J McQuay

**Affiliations:** 1 Pain Research and Nuffield Department of Anaesthetics, University of Oxford, Oxford Radcliffe Hospital, Oxford OX3 7LJ, UK; 2 Department of Rheumatology, Musgrove Park Hospital, Stockman's Lane, Belfast BT0 7JB, UK

## Abstract

**Introduction:**

Osteoarthritis trials usually report average changes in visual analogue scale (VAS) pain, and examine the difference between treatment and placebo. We investigated whether dichotomous responder analysis provides a more informative interpretation of drug efficacy.

**Methods:**

Merck supplied the number of patients who, by 6 weeks, had achieved pain relief compared with a baseline of 0% or more, 10% or more, 20% or more, and so on at equal intervals up to 90% or more. These different levels of pain relief were used to distinguish different definitions of responders, for example at least 50% pain relief from baseline. Numbers and percentages of patients achieving each level were identified. Information was sought from a dose–response trial over 6 weeks in osteoarthritis using placebo and using etoricoxib at 5, 10, 30 and 60 mg daily.

**Results:**

With placebo, the proportions of patients achieving at least 20%, 50% and 70% pain relief over baseline at 6 weeks were 30%, 11% and 2%. With 60 mg etoricoxib the equivalent percentages were 74%, 49% and 29%. The numbers needed to treat for 30 mg and 60 mg etoricoxib to produce at least 50% pain relief at 6 weeks compared with placebo were 4.2 (95% confidence interval 3.8 to 8.6) and 2.6 (2.0 to 3.9), respectively. Levels of pain relief of 50% and above discriminated best between different doses of etoricoxib.

**Conclusion:**

Responder analysis seemed to be more sensitive than examination of average changes in VAS pain scores. Validation would require calculations to be performed on a set of trials using individual patient data not available in publications.

## Introduction

In recent years, meta-analyses of randomised trials in osteoarthritis have suggested that the benefits of some well-established therapies – oral non-steroidal anti-inflammatory drugs (NSAIDs), topical NSAIDs, intra-articular steroid injections, and opioids – are small and limited to the first 2 to 3 weeks after the start of treatment [1]. The argument is that, with 10 mm out of 100 mm average difference over placebo, the benefits just reach a threshold for minimal perceptible improvement, and barely achieve the threshold for a slight improvement. Criticism of these therapies even suggests 'that it is time to reconsider the place of these drug therapies in OAK [osteoarthritis of the knee] management'.

This and previous papers [2] have been criticised on the basis that average results from clinical trials do not adequately capture benefits to individuals [3,4], and that clinical trials measure what is measurable, not necessarily what is important [4]. Considerable effort has gone into looking at ways in which outputs in arthritis trials can be made more relevant to clinical practice, for individuals as a therapeutic success [5], or by efforts to incorporate priorities from subscales [6].

Whatever the eventual success of these methods, science is informing us that there are very large differences between individuals, and that small changes in genetic makeup can greatly influence response to drugs. We know, for instance, that there is variation in plasma concentration and pharmacological response [7], and this may be responsible for some of the large differences between patients in outcomes such as blood pressure [8]. Similar issues affect morphine [9] and other analgesics [10]. In acute pain, patients also show large differences, some having virtually no pain relief whereas others have high levels of pain relief, but few patients are found to be average [11]. The use of average results from such highly skewed distributions has been shown to produce unreliable results [12]. Clinical trials in depression have investigated the individual response [13], and this has led to the assertion that 'equal on average is not equal for everyone' [14].

We therefore sought to use data from a single clinical trial in osteoarthritis to explore whether a more informative interpretation of osteoarthritis trials might be obtained by using dichotomous responder analysis, as has been done previously for acute [11,15] and chronic pain [16,17]. This was intended as a pilot analysis, which, if successful, could be extended into a more detailed examination of possible outcomes derived from dichotomous rather than continuous scores, using larger data sets and meta-analytic methods.

## Materials and methods

To obtain a range of responses, we asked Merck Research Laboratories (Rahway, NJ, USA) for responder information from clinical trial 007 [18]. They provided data on placebo and on 5, 10, 30 and 60 mg doses of etoricoxib. The trial was double blind and randomised, and included patients with radiographic and clinical diagnosis of knee osteoarthritis who were at least 40 years old and whose symptoms had persisted for at least 6 months.

Patients discontinued their pre-study NSAID for a period ranging from 3 to 8 days (for instance diclofenac) to 10 to 15 days (piroxicam). For inclusion, pain on a 100 mm scale had to be a minimum 40 mm walking on a flat surface at the flare visit, plus at least 15 mm increase and worsening in investigator global assessment since baseline visit. This was designated the flare, and if patients fulfilled these and other criteria they were randomised to treatment with placebo (n = 60) or with etoricoxib at single daily doses of 5 mg (n = 117), 10 mg (n = 114), 30 mg (n = 102), 60 mg (n = 112) or 90 mg (n = 112) for 6 weeks.

We asked Merck to supply the number of patients in each group who, by 6 weeks, had achieved pain relief compared with baseline of at least 0%, at least 10%, at least 20%, and so on at equal points to at least 90%. The numbers and percentages of patients achieving, say, at least 50% pain relief from baseline, might be defined as a responder, and presentation of data in this way allowed different definitions of responder to be applied.

The number needed to treat (NNT) to produce each level of response for each etoricoxib dose compared with placebo was calculated, with 95% confidence interval (CI) [19]. Relative risk with 95% CI was calculated by using the fixed effects model [20] and was considered to be statistically significant when the 95% CI did not include one.

## Results

Patients in the trial were predominantly female (72%) and white (89%), were aged between 40 and 87 years, had a median duration of arthritis of 6 years, and were mostly diagnosed as American Rheumatism Association class II/III (85%). Most patients completed 6 weeks of therapy, with all-cause discontinuation rates of 8 to 17% in different groups.

**Figure 1:**
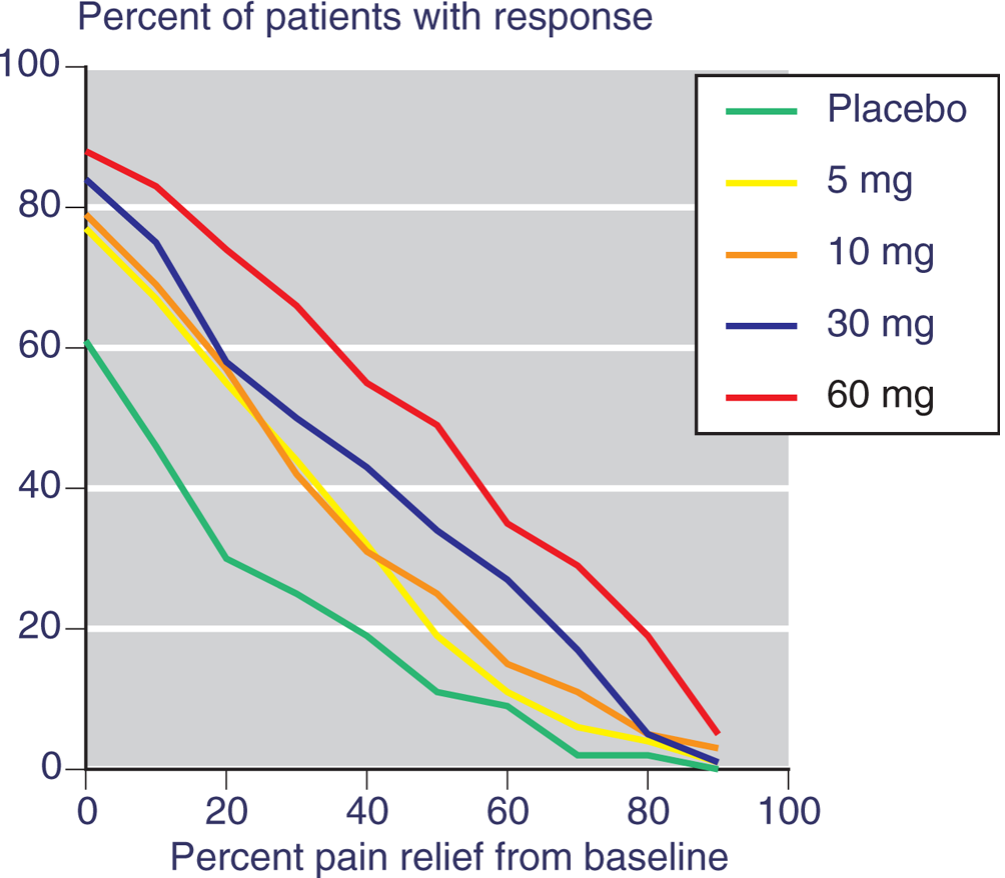
Percentage of patients achieving various levels of pain relief at 6 weeks compared with baseline.

There was a very wide individual range of responses to the various treatments. With each, some patients achieved only small amounts of pain relief, whereas others had close to complete relief. Table 1 shows the percentage of patients in each treatment group who achieved various levels of pain relief at 6 weeks compared with baseline, taking placebo or 5, 10, 30 or 60 mg etoricoxib. Data on the 90 mg dose was not made available. Figure 1 shows how the percentage of patients defined as responders at each level of pain relief declined with increasing levels of pain relief. Whereas 30% of patients achieved at least 20% pain relief with placebo, only 11% achieved at least 50% pain relief, and only 2% achieved at least 70% pain relief. For 60 mg etoricoxib, 74% achieved at least 20% pain relief, 49% at least 50%, and 29% at least 70%.

**Table 1:** Percentage of patients group achieving various levels of pain relief at 6 weeks compared with baseline

Percentage reduction in pain from baseline	Treatment group: etoricoxib daily dose (mg)				
	
	0 (placebo) (n = 57)	5 (n = 114)	10 (n = 105)	30 (n = 102)	60 (n = 109)
>0	61	77	79	84	88
≥ 10	46	67	69	75	83
≥ 20	30	55	57	58	74
≥ 30	25	44	42	50	66
≥ 40	19	32	31	43	55
≥ 50	11	19	25	34	49
≥ 60	9	11	15	27	35
≥ 70	2	6	11	17	29
≥ 80	2	4	5	5	19
≥ 90	0	1	3	1	5

Using at least 50% pain relief as an arbitrary level of success to define response, the absolute differences between placebo and 30 and 60 mg etoricoxib daily at 6 weeks were 24% and 38% of patients, respectively. The NNTs for 30 and 60 mg etoricoxib to produce at least 50% pain relief at 6 weeks compared with placebo were 4.2 (95% CI 3.8 to 8.6) and 2.6 (2.0 to 3.9), respectively.

**Figure 2:**
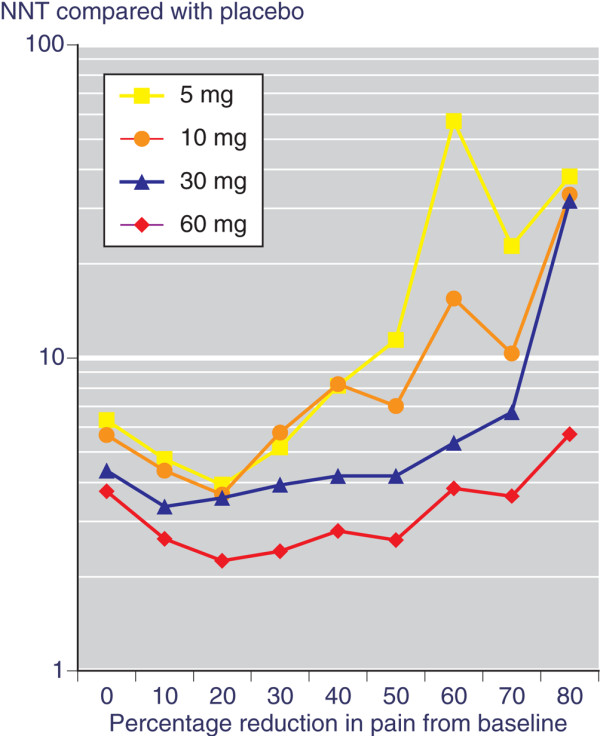
Number needed to treat for each dose of etoricoxib at each level of pain relief. NNT, number needed to treat.

The absolute differences between etoricoxib and placebo were used to calculate NNTs at each level of pain relief (Figure 2). At lower levels of pain relief there was limited discrimination between the different doses, but at higher levels there was greater discrimination. A level of at least 50% pain relief from baseline at 6 weeks produced an obvious dose response. Higher levels of pain relief resulted in higher (worse) NNTs for 5 and 10 mg etoricoxib, while the 30 and 60 mg etoricoxib doses maintained stable and reasonably low (good) NNTs over the range of at least 10% pain relief to at least 70% pain relief (range of NNTs 3.5 to 6.7 for 30 mg, and 2.3 to 3.6 for 60 mg).

## Discussion

The trial [18] originally reported mean differences over placebo of the same order as the Bjordal meta-analysis [1]. They were 8 mm (5 mg), 10 mm (10 mg), 14 mm (30 mg), 22 mm (60 mg) and 19 mm (90 mg) on a 100 mm visual analogue scale (VAS). We have used the same information from the same trial in the form of a responder analysis to examine whether such an analysis is more informative. The responder analysis demonstrated that a larger proportion of patients achieved higher levels of pain relief with active treatment than with placebo (Figure 1), and that the absolute difference, illustrated by the NNT, was large, clinically significant, and more discriminatory at higher levels of pain relief (Figure 2).

This is not a surprise. Although fewer than half of the patients achieved at least 50% pain relief with 60 mg etoricoxib, this level of response is not uncommon. For instance, in migraine it is common for oral drugs to yield 50 to 60% response rates with the low hurdle of no pain or mild pain at 2 hours after an attack, but this falls to 20 to 40% for pain-free at 2 hours [21]. In neuropathic pain fewer than half of patients achieve 50% pain relief with duloxetine [17], and about half with higher doses of pregabalin [16]. Proportions were even lower in breakthrough pain treatment [16]. In acute pain trials in standardised pain models, commonly used drug and dose combinations typically produce response rates of 40 to 60% [22], and deeper analysis shows that patients are either responders or not [11]. Lower response rates are seen in the treatment of depression [13]. Genetics argues for considerable inter-individual responses to drugs [7], leading to limited response rates for any particular drug.

The differences between active drug and placebo were large. For instance, the NNTs for at least 50% pain relief of 4.2 (95% CI 3.8 to 8.6) and 2.6 (2.0 to 3.9) for 30 and 60 mg etoricoxib in osteoarthritis compare well with those found for at least 50% pain relief in postoperative pain (range 2 to more than 6 [22]), migraine (2.6 to 5.4 [21]), and neuropathic pain (2.6 to more than 8 [23]). For a 50% improvement in symptoms according to the American College of Rheumatology criteria (ACR50) after 12 months of therapy, NNTs of 4 were recorded with adalimumab, etenercept and double-dose infliximab [24]. These examples of NNTs from other painful conditions have similar outcomes, if different timescales. Although no direct comparison is possible, NNTs of 5 and below are generally regarded as markers of effective treatment, but much higher values are useful for some prophylactic interventions [25].

Greater discrimination between pain therapies at higher levels of pain relief has been shown previously for acute pain [15]. That better therapies should result in more patients with higher levels of relief is not surprising, but individual patient analysis has not been done for migraine or neuropathic pain to allow a comparison to be made.

Other workers have attempted to calculate numbers needed to treat for osteoarthritis trials. For example, NNTs of 3 to 4 were calculated for pain reduction and patient global assessment after intra-articular corticosteroid, on the basis of fewer than 200 patients [26]. The NNT to achieve improvement in pain ranged from 4 to 16 in an analysis of acetaminophen in osteoarthritis [27]. The results calculated in this paper by using the Western Ontario McMaster (WOMAC) pain scale were at least comparable.

This exploratory study is limited by size, and by examining only one trial. Validation would require calculations to be performed on a larger set of trials using individual patient data not available in publications, and expanded to scales other than pain while walking on a flat surface. Outcomes other than pain might be considered, particularly the OMERACT-OARSI (outcome measures in rheumatoid arthritis clinical trials of the Osteoarthritis Research Society International) definition of responder (defined as a patient with at least 50% improvement in pain or function that was at least 20 mm on a 100 mm VAS, or at least 20% improvement in at least two of pain, function or patient global assessment that was at least 10 mm on a 100 mm VAS [28]).

It would also be possible to test the discriminating power of various outcomes in larger, better-conducted trials. It is maintained that dichotomous outcomes have less statistical power than continuous outcomes [29]. This has been demonstrated for studies in which the sample size is small [30]. That may not, however, always be so, and a well-defined dichotomous outcome can approach or exceed the power of a continuous outcome [31].

The question is: What makes a good definition of improvement for a patient in a clinical trial? Any definition should embody truth, discrimination and feasibility, and so it should be readily translatable for use in a clinical trial, make clinical sense, be specific to the clinical situation, have good statistical power, and be easy to calculate and interpret [31]. It is not necessarily true that what makes best statistical sense or utility is what is best for describing possible outcomes for patients, including benefits alongside risks [32].

Average differences in visual analogue pain scales between active drugs and placebo in arthritis trials seem to understate the efficacy of active medicines. Dichotomous responder analysis using higher levels of pain relief of at least 50% over baseline demonstrated an efficacy equivalent to that measured in other pain states, contradicting the idea that that it is time to reconsider the place of drug therapies in arthritis [1].

## Conclusion

Reporting of a responder analysis, called a cumulative proportion of responders analysis, as well as the actual proportions achieving certain levels of pain relief (30%, 50% and 70%, say) may be an important addition to clinical trial reporting. It helps to show potential benefits of higher and lower than average doses for individual patients, and possibly highlights different criteria for determining effective or licensed doses. This is much more informative than average differences in VAS pain between treatment and placebo.

## Abbreviations

CI = confidence interval; NNT = number needed to treat; NSAID = non-steroidal anti-inflammatory drug; VAS = visual analogue scale.

## Competing interests

RAM and HJM have received research grants, consulting fees or lecture fees from pharmaceutical companies, including Pfizer, MSD, GSK, AstraZeneca, Grunenthal, Menarini and Futura. RAM, HJM and SD have also received research support from charities and government sources at various times. RAM is the guarantor. No author has any direct stock holding in any pharmaceutical company.

## Authors' contributions

RAM was involved with the original concept, planning the study, searching, writing it, analysis, and preparing the manuscript; OAM and RAM performed calculations and analysis; OAM, SD and HJM were involved with planning and writing. All authors read and approved the final manuscript.
